# Complete Remission of Vocal Cord Cancer Treated With Low-Dose Ipilimumab Plus Nivolumab Combined With Interleukin-2 and Hyperthermia

**DOI:** 10.7759/cureus.14500

**Published:** 2021-04-15

**Authors:** Ralf Kleef, Viktor Bacher, Robert Nagy, Peter Reisegger, Tibor Bakacs

**Affiliations:** 1 Immunology & Integrative Oncology, Dr. Kleef Hyperthermia, Vienna, AUT; 2 ENT, ENT Specialist, Vienna, AUT; 3 Probability, Alfred Renyi Institute of Mathematics; The Eötvös Loránd Research Network (ELKH), Budapest, HUN

**Keywords:** advanced cancer, vocal cord cancer, immunotherapy, hyperthermia, il-2, checkpoint inhibitors, iraes, recurrence local, head and neck squamous cell carcinoma (hnscc), cordectomy

## Abstract

We present a 44-year-old male patient, exposed to tobacco smoke and alcohol, with a locally advanced, multiple recurrent squamous cell carcinoma (SCC) of the vocal cord who had undergone resection four times. The patient rejected the mutilating surgery or radiation therapy due to the expected severe lifelong consequences. Instead, the patient opted for complex immunotherapy combining low doses of checkpoint inhibitors ipilimumab-nivolumab (0.3 and 0.5 mg/kg, respectively) with fever-inducing interleukin-2 (IL-2) and hyperthermia, which induced complete remission (CR). Restaging with MRI and laryngoscopy demonstrated lasting remission ongoing now for two years. The fact that this patient is free of any cancer-related signs or symptoms raises the possibility of a long-lasting remission even after the fourth recurrence of a locally advanced squamous cell vocal cord cancer by the induction of therapeutic fever combined with a safe low-dose ipilimumab plus nivolumab therapy to endorse T-cell function.

## Introduction

The Global Cancer Report announced in 2018 that head and neck squamous cell carcinoma (HNSCC) was the eighth most frequent cancer. Its mortality rate ranked eighth of all cancers [1]. Despite improved survival rates for cancer patients over the past 20 years, failure of local and distant treatment of advanced HNSCC occurs in up to 40% and 30% of patients, respectively [2]. Vocal cord cancer is very closely linked with a history of smoking, though nonsmokers may also get this cancer. Many vocal cord cancers present early because the lesion creates hoarseness that often prompts early evaluation and early treatment can often induce a lasting remission. However, the case presented here was too advanced for curing without major disabling consequences. Despite the fact that immunotherapy with checkpoint inhibitors is licensed for head and neck cancer, this patient was offered only total laryngectomy or high-dose radiation.

The Society for Immunotherapy of Cancer (SITC) formed an expert committee to work out consensus recommendations for emerging immunotherapies in different cancer types including head and neck cancer. The consensus guidelines assist clinicians' understanding of the role of immunotherapies in this disease setting and standardize utilization across the field for the patient benefit [3]. Because of the published evidence specifically for squamous cell cancers of the head and neck expressing PD-L1 we decided to offer immunotherapy in an experimental setting to this patient as described below.

Although case reports lack statistical sampling they provide individual clinical insights that are missed in clinical trials [4]. Consistent with this, the number of peer-reviewed journals publishing case reports has recently increased to more than 160 [5]. In fact, several breakthrough cases paved the way for revolutionary medical advances, such as, for example, the first advanced leukemia patient who was cured by the experimental chimeric antigen receptor (CAR) T cell therapy [6], or the first sickle cell disease patient who was thriving one year after the administration of the revolutionary gene-editing technique called CRISPR [7].

## Case presentation

**Figure 1:**
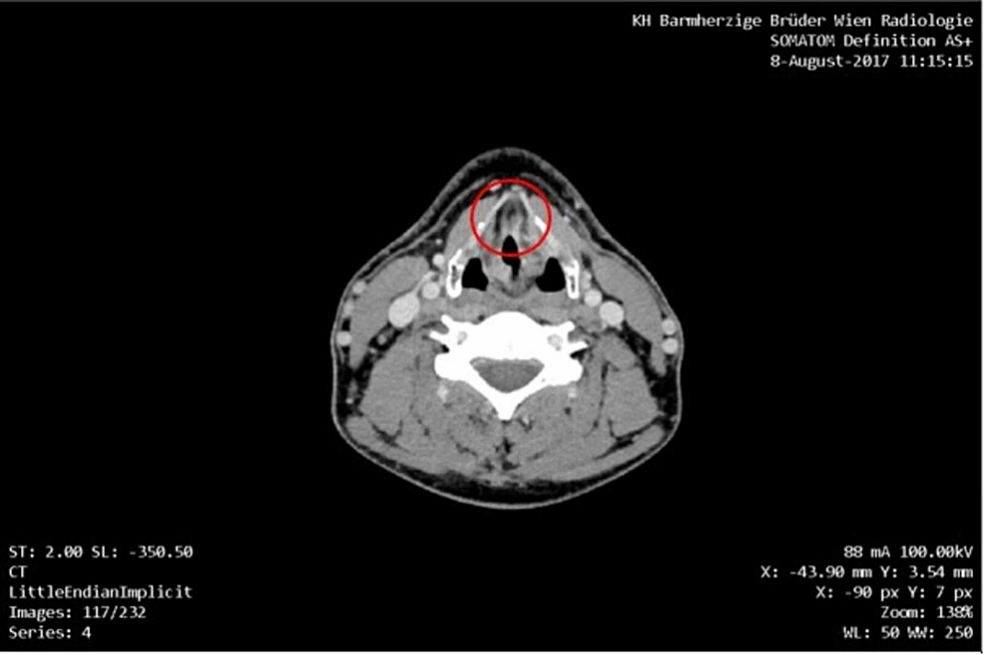
CT scan with contrast agent from August 2017. Hypodense formation (11 mm x 8 mm x 13 mm) at the commissura anterior of the vocal cord on the left, showing the recurrent tumor. No suspect lymph nodes, no lung metastases in the upper thorax, and no osseus metastases.

This 44-year-old patient was a professional DJ and exposed to tobacco smoke, alcohol and had a severely disturbed circadian rhythm. The squamous cell carcinoma (SCC) of the left vocal cord was diagnosed in 2012 when the patient experienced persisting hoarseness. According to the initial American Joint Committee on Cancer (AJCC) assessment, this was then a stage I disease. The patient underwent left side cordectomy with R0 (T1 N0 M0 L0 V0), this was followed by a watchful waiting strategy for one year. The first local recurrence occurred in 2013 treated with R0 resection again (T1a N0 M0 L0 V0). The second local recurrence occurred in February 2017 and was treated by extended left-sided cordectomy type Va R0 (this time T2 N0 M0 L0 V0). By now the tumor became AJCC stage II. In March 2017, post-resection surgery was performed due to complications. In August 2017, the third local recurrence sized 13 mm x 11 mm x 8 mm was demonstrated by pan-endoscopy and CT of the neck, abdomen, and chest (Figure 1).

**Figure 2:**
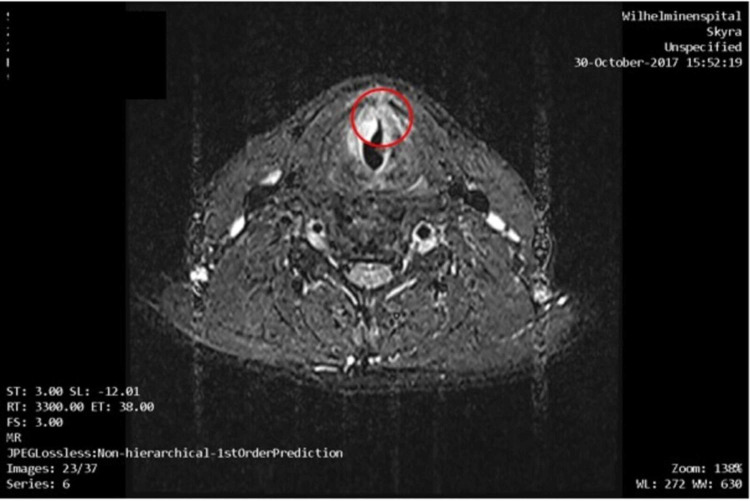
MRI with contrast agent from October 2017. Soft tissue formation (13 mm x 9 mm x 13 mm) at the commissura anterior of the vocal cord on the left with contrast enhancement and diffusion restriction, showing the recurrent tumor, approximately the same size as in previously performed CT. Additionally, there is a bilateral swelling of the vocal cords, caused by the tumor with progressive narrowing of the airways in comparison to the previously performed CT. Lymph nodes increased in number and borderline in size in Level 1b, 2, and 3 on both sides.

By now the tumor became AJCC stage III (T3 N1 M0 L1 V0). MRI described the lesion as 10 mm × 18 mm (Figure 2).

The patient underwent repeated biopsy demonstrating recurrence of a nonkeratinizing, low differentiated SCC G3 in the left and right anterior commissure. The patient was warned of acute life-threatening danger since due to the narrowing of the upper airways his life expectancy (without intervention) would be between four and six weeks. After this third recurrence no specific interventions were carried out, only complementary medicine was applied, including dichloroacetate (DCA), curcumin, and methadone treatments. Further laboratory analysis revealed elevated tumor markers M2PK (62,9), and SCC Ag 0.7 ng/mL.

To resolve the progressive shortness of breath caused by the inoperable tumor either total laryngectomy or primary radiation with 70 Gy including the cervical lymphatic system were offered. The patient, however, refused mutilating surgery or radiation given the expected severe lifelong debilitating consequences.

**Figure 3:**
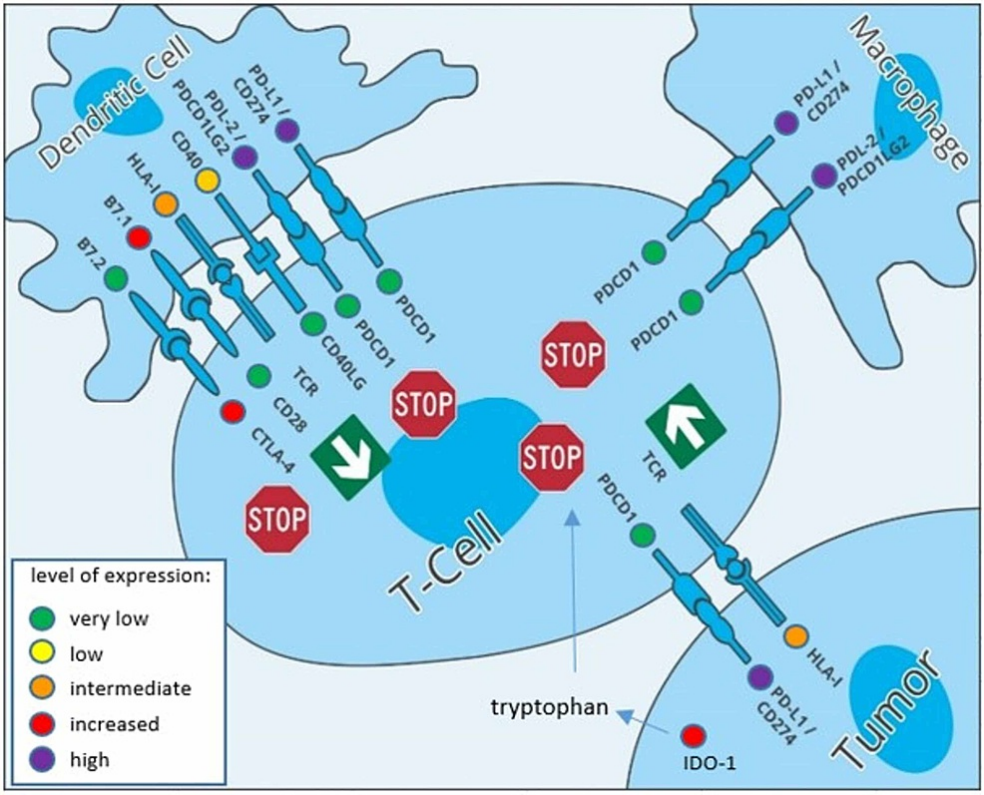
mRNA expression analysis of the tumor micro-environment. The figure displays high expression of PD-L1 and PD-L2 (violet color) correlating to an immunosuppressive micro-environment. These ligands are promising targets for ICIs. The increased expression of IDO-1 (red color) is also associated with mediating potently immunosuppressive effects in cancer and reduced T-cell infiltration, which is here implicated by low detection level of T-cell markers (green color). ICIs, immune checkpoint inhibitors *Courtesy*: NextGen Oncology Group Dusseldorf, Germany. Data are based on array hybridization.

The patient presented at our clinic in August 2017 for the first time with a Karnofsky-index of 90%, ECOG 1 in good overall condition. He had very strong hoarseness, dysphonia, and some difficulties swallowing solid food. He had a negative family history of cancer. Following exact staging with CT, MRI, and video endoscopy, the tumor tissue was analyzed by next-generation sequencing (NGS), which was complemented by additional immunohistochemical analyses. The 44 K Chip of Agilent Technologies, comprising 44,000 genetic probes was used. The activity of all human genes (approximately 21,000) in the tumor tissue using >34,000 gene expression markers was examined. Approximately 1000 genes with high clinical relevance were present as 10-fold replicates. The interpretation of NGS analysis is based on publicly available gene signatures (Figure 3).

Immune histochemistry revealed a high elevation of proliferation marker MIB1/Ki-67 of 90%, EGFR neg., p53 positive mutated, and expression of 6 relevant checkpoint genes IDO1 57%, CD40 56%, PD-L1 96%, CTLA-4 65%, LAG3 68%, PD-L2 95%.

Therapy began in September 2017 with local hyperthermia treatment. During this period the patient received nine treatments with the Oncotherm® (Oncotherm Kft, Budaörs, Hungary) 13.56 MHz device applied locally on the left side of the neck with the smallest radiofrequency applicator. The loco-regional hyperthermia was complemented with a high dose (0.5 g/kg) IV vitamin C and alpha-lipoic acid 600 mg treatment three times weekly.

In January 2018, the patient received a combined immune therapy, the Kleef-protocol [8] including loco-regional radiofrequency (13.56 MHz) hyperthermia (Oncotherm EHY2000 device) and whole-body hyperthermia (Heckel HT3000 device, Heckel Medizintechnik GmbH, Esslingen, Germany), low-dose combined checkpoint inhibitors (ipilimumab plus nivolumab), and individually titrated IL-2. Ipilimumab and nivolumab were dosed with 0.3 and 0.5 mg/kg, respectively, weekly over three weeks. IL-2 (Proleukin®, Clinigen Healthcare Ltd., Staffordshire, United Kingdom) was carefully administered with continuous temperature measurement on the monitor to achieve fever range temperatures. Immune-related adverse events (irAEs) regarding local tumor pain were grade III directly during treatment but vanished soon after. It is important to note that local pain and hoarseness increased transiently during the treatment probably due to the expected inflammatory reaction within the tumor microenvironment. These symptoms rapidly vanished following the termination of the treatment. Fever following induction of IL-2 therapy should not be viewed as an irAE because it was deliberately induced under strict clinical monitoring up to 40°C. No other serious irAEs were observed.

**Figure 4:**
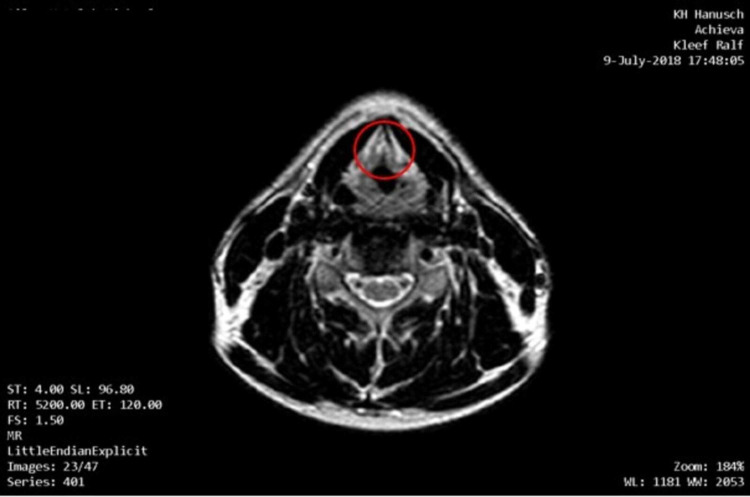
MRI with contrast agent from July 2018. After therapy regular configuration of vocal cords and commissura anterior, no detectable tumor tissue, no signs of edema, showing complete remission.

**Figure 5:**
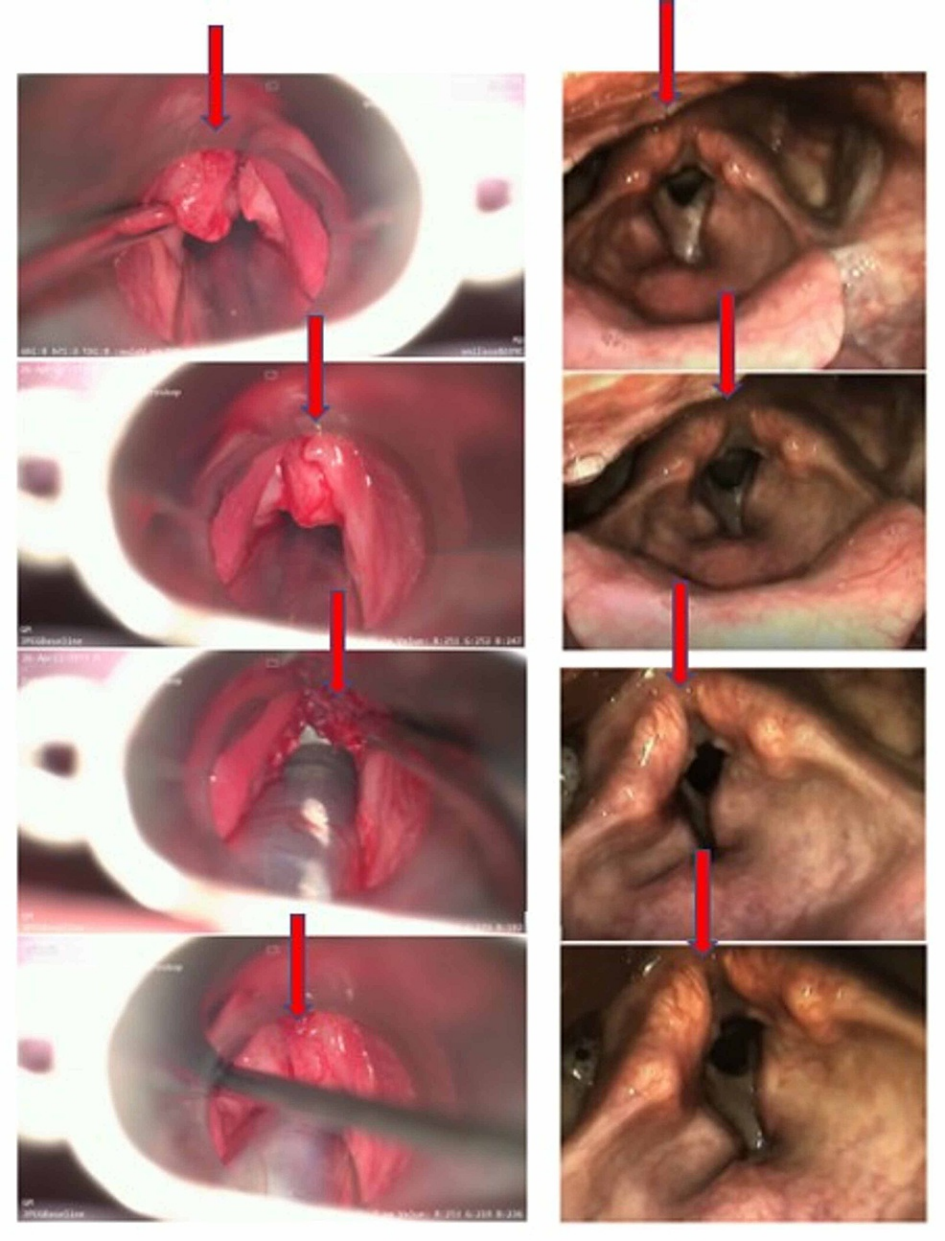
Comparative pictures taken during video laryngoscopy in August 2017 versus August 2020. The third recurrence of the primary tumor on the left (2017) is in remarkable CR on the right (2020). CR, complete remission

Initial MRI in February 2018 showed signs of the stabilization of the disease (SD). Repeated follow-up with MRI of the neck (Figure 4) and PET-CT, as well as repeated laryngoscopic examination, revealed complete remission (CR) already in July 2018. Last laryngoscopic examination in December 2020 demonstrated ongoing CR (Figure 5). 

.

## Discussion

The combined immune therapy approach include both loco-regional and whole-body hyperthermia, low-dose checkpoint inhibitors, and individually dosed IL-2 induced CR of the locally advanced and multiple times recurrent squamous cell cancer of the vocal cord. Restaging with MRI and laryngoscopy demonstrates lasting remission, which is ongoing for two years. The patient is free of any cancer-related signs or symptoms. This CR, in our view, would not have been possible without the administration of an off-label low-dose concurrent ipilimumab and nivolumab immune checkpoint inhibitor (ICI) therapy.

In 2018, a special issue of Science reviewed the newly approved immunotherapies that led to a clinical breakthrough via the translation of ICIs by manipulating components of the immune system to attack tumors [9]. Unfortunately, such manipulation has also resulted in a major safety issue: iatrogenic irAEs. While concurrent ipilimumab and nivolumab ICI therapy provide a long-lasting remission in the majority of patients with advanced melanoma, irAEs were reported in 97% of patients, 59% of which were grade 3 and 4 leading to discontinuation in 25% of patients and one death [10]. Actually, the deleterious effects of severe irAEs might outweigh the benefit from the addition of ipilimumab [11].

In the above context, it was a very significant progress that we demonstrated in 131 unselected stage IV cancer patients with 23 different cancer types who were treated with the Kleef-protocol [8], an objective response rate of 31.3%, progression-free survival of 10 months, and survival-probabilities of 66.5% at 12 months. The irAEs of World Health Organization (WHO) Toxicity Scale grade 3 and 4 were observed in only 8.4% of patients suggesting that the combined treatment is safer than the established protocols without compromising efficacy. Actually, our protocol does not require the selection of stage IV cancer patients who are more likely to gain benefit from ICIs [12]. As a matter of fact, our stage IV patients had several negative pre-selection factors such as antibiotic use in 24%, low PD-1/PD-L1 expression, 35.1% of patients had liver metastasis, which is associated with a specifically bad prognosis, and only 35% of the patients had ECOG 0, while all the patients were heavily pretreated. Despite these facts, 15.3% of the patients achieved a complete response with 20.7 months median time until progression [8]. The presented case is one of them demonstrating the potential of this new combination therapy.

Importantly, Kleef’s approach takes advantage of mechanisms proposed in several immune models, including the DAMP model that needs for signals triggered by damage/danger-associated molecular patterns, the coinhibition model which is based on the assumption that negative feedback mediated by coinhibitory signals generates peripheral tolerance, and the quorum model which proposes that the adaptive immune system uses an antigen-specific counting mechanism [13]. Since the Kleef-protocol [8] consists only of approved drugs and treatments, our hypothesis that autoimmune T cells induced by a safe low‐dose ICI therapy in combination with external hyperthermia and internal hyperthermia (IL-2) are powerful therapeutic tools can be confirmed or refuted in controlled prospective clinical trials.

## Conclusions

The presented case is currently out from his diagnosis of inoperable advanced disease for two years without any evidence of persistent or recurrent cancer. This case raises the possibility of a long-lasting remission even for stage IV disease by the induction of therapeutic fever combined with a safe low-dose ipilimumab (0.3 mg/kg) and nivolumab (0.5 mg/kg) therapy to endorse T-cell function. By presenting all relevant details of CR in stage IV cancer we hope to achieve a broader clinical impact across medicine.
